# Bis[dieth­yl(hy­droxy)ammonium] benzene-1,4-dicarboxyl­ate

**DOI:** 10.1107/S1600536810027868

**Published:** 2010-07-21

**Authors:** De-Ming Xie, Chun-Xiao Chen, Hai-Xiang Chen, Hai-Bin Wang

**Affiliations:** a Research Institute of Materials and Surface Engineering, School of Mechnical Engineering, Zhejiang University of Technology, Hangzhou 310032, People’s Republic of China; b Analytical Center, Zhejiang Sci-Tech University, Hangzhou 310018, People’s Republic of China; c College of Chemical Engineering and Materials Science, Zhejiang University of Technology, Hangzhou 310018, People’s Republic of China

## Abstract

In the centrosymmetric title compound, 2C_4_H_12_NO^+^·C_8_H_4_O_4_^2−^, two *N*,*N*-dieth­yl(hy­droxy)ammonium cations are linked to a benzene-1,4-dicarboxyl­ate dianion by a combination of O—H⋯O and N—H⋯O hydrogen bonds, which can be described in graph-set terminology as *R*_2_^2^(7). The crystal structure is further stabilized by C—H⋯O hydrogen bonds, leading to the fomation of a ribbon-like network.

## Related literature

For similar supamolecular structures involving benzene­dicarb­oxy­lic acids, see: Chatterjee *et al.* (2000 ▶); Herbstein & Kapon (1978 ▶); Karpova *et al.* (2004 ▶); Mak & Xue (2000 ▶); Yuge *et al.* (2006 ▶); Zhao *et al.* (2007 ▶). For graph-set theory, see: Bernstein *et al.* (1995 ▶).
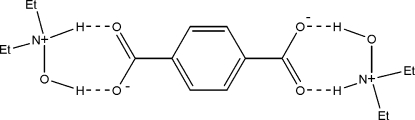


## Experimental

### 

#### Crystal data

2C_4_H_12_NO^+^·C_8_H_4_O_4_^2−^
*M* *_r_* = 344.40
Monoclinic, 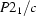

*a* = 6.507 (2) Å
*b* = 11.478 (4) Å
*c* = 12.649 (5) Å
β = 97.380 (7)°
*V* = 936.9 (6) Å^3^
*Z* = 2
Mo *K*α radiation
μ = 0.09 mm^−1^
*T* = 273 K
0.37 × 0.31 × 0.27 mm

#### Data collection

Bruker SMART CCD area-detector diffractometer
Absorption correction: multi-scan (*SADABS*; Bruker, 2007 ▶) *T*_min_ = 0.954, *T*_max_ = 0.969
4737 measured reflections
1653 independent reflections
1460 reflections with *I* > 2σ(*I*)
*R*_int_ = 0.023

#### Refinement

*R*[*F* ^2^ > 2σ(*F* ^2^)] = 0.087
*wR*(*F* ^2^) = 0.220
*S* = 1.13
1653 reflections
112 parameters
H-atom parameters constrained
Δρ_max_ = 0.66 e Å^−3^
Δρ_min_ = −0.27 e Å^−3^

### 

Data collection: *SMART* (Bruker, 2007 ▶); cell refinement: *SAINT* (Bruker, 2007 ▶); data reduction: *SAINT*; program(s) used to solve structure: *SHELXS97* (Sheldrick, 2008 ▶); program(s) used to refine structure: *SHELXL97* (Sheldrick, 2008 ▶); molecular graphics: *SHELXTL* (Sheldrick, 2008 ▶); software used to prepare material for publication: *SHELXTL*.

## Supplementary Material

Crystal structure: contains datablocks global, I. DOI: 10.1107/S1600536810027868/su2169sup1.cif

Structure factors: contains datablocks I. DOI: 10.1107/S1600536810027868/su2169Isup2.hkl

Additional supplementary materials:  crystallographic information; 3D view; checkCIF report

## Figures and Tables

**Table 1:** Hydrogen-bond geometry (Å, °)

*D*—H⋯*A*	*D*—H	H⋯*A*	*D*⋯*A*	*D*—H⋯*A*
O3—H3⋯O2	0.82	1.78	2.576 (5)	164
N1—H1⋯O1	0.91	1.72	2.605 (5)	164
C7—H7b⋯O2^i^	0.97	2.42	3.327 (5)	156

Symmetry code: (i) 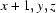.

**Fig. 1.**
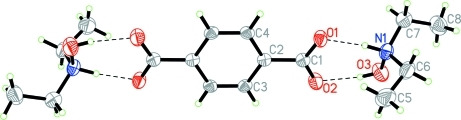
A view of the molecular structure of the title compound [The O—H···O and N—H···O hydrogen bonds are illustrated by dotted lines].

**Fig. 2.**
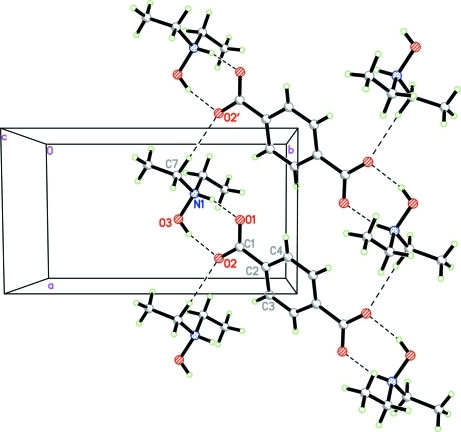
A perspective view, along the c-axis, of the crystal packing of the title compound [The O—H···O and N—H···O hydrogen bonds nd the C-H···O interactions are illustrated by dotted lines; the symmetry code for the atom labeled O2' is = x+1, y, z].

## supplementary crystallographic information

### Comment

Supramolecular aggregate design is an active field of research and in a series of papers various supramolecular structures comprising benzene-dicarboxylic acids have been elucidated (Herbstein *et al.*, 1978; Chatterjee *et al.*, 2000; Karpova *et al.*, 2004; Zhao *et al.*, 2007). Some cases have been reported where the use of terephthalic acid has lead to the fomation of supramolecular architectures through hydrogen bonding (Mak *et al.*, 2000; Yuge *et al.*, 2006). The title compound was synthesized by the reaction of terephthalic acid with N,*N*-diethylhydroxylammine.

As shown in Fig. 1 two *N*,*N*-diethylhydroxylammonium (DTHA) cations are linked to the benzene-1,4-dicarboxylate anion (BDL), which is situated about an inversion center, by a special combination of O—H···O and N—H···O hydrogen bonds (Table 1), N1—H1···O1 and O3—H3···O2, which can be described by graph-set R^2^_2_(7) [Bernstein, *et al.*, 1995].

In the BDL anion the dihedral angle between phenyl ring and carboxylate group is 11.3 (3)/%. In general the BDLanion is almost coplanar with the mean plane through the C and N-atoms in the DTHA cations. The carboxylate groups are nearly perpendicular with the mean plane through the C and N-atoms of DTHA [dihedral angle of 81.0 (3)/%].

In the crystal structure a ribbon-like structure (Fig. 2 and Table 1), is fomed via C7—H7···O2^i^ interactions [symmetry code (i) = 1 + x, y, z] .

### Experimental

N,*N*-diethylhydroxylammine and terephthalic acid, in a molar ratio of 2:1, were mixed and dissolved in sufficient ethanol that by heating to 353 K a clear solution was obtained. The reaction system was then cooled slowly to RT, and crystals of the title compound were formed. They were collected and washed with ethanol.

### Refinement

The H-atoms were included in calculated positions and treated as riding atoms: O-H = 0.82 Å, N-H = 0.91 Å, C-H = 0.93, 0.96, and 0.97 Å for aromatic, methyl and methylene H-atoms, respectively, with U_iso_(H) = k × U_eq_(parent O, N or C atom), where k = 1.5 for hydroxyl and methyl H-atoms and = 1.2 for all others.

### Crystal data

**Table:**

2C_4_H_12_NO^+^·C_8_H_4_O_4_^2^^−^	*F*(000) = 372.0
*M**_r_* = 344.40	*D*_x_ = 1.221 Mg m^−^^3^
Monoclinic, *P*2_1_/*c*	Mo *K*α radiation, λ = 0.71073 Å
Hall symbol: -P 2ybc	Cell parameters from 2185 reflections
*a* = 6.507 (2) Å	θ = 2.1–25.0°
*b* = 11.478 (4) Å	µ = 0.09 mm^−^^1^
*c* = 12.649 (5) Å	*T* = 273 K
β = 97.380 (7)°	Block, colorless
*V* = 936.9 (6) Å^3^	0.37 × 0.31 × 0.27 mm
*Z* = 2	

### Data collection

**Table:**

Bruker SMART CCD area-detector diffractometer	1653 independent reflections
Radiation source: fine-focus sealed tube	1460 reflections with *I* > 2σ(*I*)
graphite	*R*_int_ = 0.023
phi and ω scans	θ_max_ = 25.0°, θ_min_ = 2.4°
Absorption correction: multi-scan (*SADABS*; Bruker, 2007)	*h* = −7→7
*T*_min_ = 0.954, *T*_max_ = 0.969	*k* = −10→13
4737 measured reflections	*l* = −14→15

### Refinement

**Table:**

Refinement on *F*^2^	Primary atom site location: structure-invariant direct methods
Least-squares matrix: full	Secondary atom site location: difference Fourier map
*R*[*F*^2^ > 2σ(*F*^2^)] = 0.087	Hydrogen site location: inferred from neighbouring sites
*wR*(*F*^2^) = 0.220	H-atom parameters constrained
*S* = 1.13	*w* = 1/[σ^2^(*F*_o_^2^) + (0.0871*P*)^2^ + 0.9891*P*] where *P* = (*F*_o_^2^ + 2*F*_c_^2^)/3
1653 reflections	(Δ/σ)_max_ < 0.001
112 parameters	Δρ_max_ = 0.66 e Å^−^^3^
0 restraints	Δρ_min_ = −0.27 e Å^−^^3^

### Special details

**Table:**

Geometry. All esds (except the esd in the dihedral angle between two l.s. planes) are estimated using the full covariance matrix. The cell esds are taken into account individually in the estimation of esds in distances, angles and torsion angles; correlations between esds in cell parameters are only used when they are defined by crystal symmetry. An approximate (isotropic) treatment of cell esds is used for estimating esds involving l.s. planes.
Refinement. Refinement of *F*^2^ against ALL reflections. The weighted *R*-factor wR and goodness of fit *S* are based on *F*^2^, conventional *R*-factors *R* are based on *F*, with *F* set to zero for negative *F*^2^. The threshold expression of *F*^2^ > σ(*F*^2^) is used only for calculating *R*-factors(gt) etc. and is not relevant to the choice of reflections for refinement. *R*-factors based on *F*^2^ are statistically about twice as large as those based on *F*, and *R*- factors based on ALL data will be even larger.

### Fractional atomic coordinates and isotropic or equivalent isotropic displacement parameters (Å^2^)

**Table:**

	*x*	*y*	*z*	*U*_iso_*/*U*_eq_	
O1	0.4427 (3)	0.3065 (2)	0.0657 (2)	0.0636 (8)	
O2	0.1752 (4)	0.2241 (2)	0.1261 (2)	0.0682 (8)	
C1	0.2564 (5)	0.3044 (3)	0.0788 (3)	0.0482 (8)	
C2	0.1228 (4)	0.4052 (3)	0.0370 (2)	0.0423 (8)	
C3	−0.0744 (5)	0.4198 (3)	0.0657 (3)	0.0476 (8)	
H3A	−0.1258	0.3653	0.1100	0.057*	
C4	0.1945 (5)	0.4869 (3)	−0.0298 (3)	0.0469 (8)	
H4	0.3257	0.4784	−0.0505	0.056*	
O3	0.4471 (5)	0.0641 (2)	0.1778 (3)	0.0851 (10)	
H3	0.3444	0.1052	0.1629	0.128*	
N1	0.6262 (5)	0.1284 (3)	0.1645 (2)	0.0573 (8)	
H1	0.5847	0.1967	0.1320	0.069*	
C5	0.6600 (11)	0.2447 (5)	0.3266 (4)	0.110 (2)	
H5A	0.6382	0.3143	0.2846	0.164*	
H5B	0.7486	0.2617	0.3914	0.164*	
H5C	0.5292	0.2162	0.3433	0.164*	
C6	0.7561 (8)	0.1574 (4)	0.2672 (3)	0.0784 (13)	
H6A	0.7786	0.0874	0.3101	0.094*	
H6B	0.8901	0.1855	0.2525	0.094*	
C7	0.7465 (6)	0.0633 (4)	0.0917 (3)	0.0660 (11)	
H7A	0.6599	0.0520	0.0241	0.079*	
H7B	0.8644	0.1102	0.0783	0.079*	
C8	0.8225 (8)	−0.0519 (4)	0.1328 (4)	0.0938 (16)	
H8A	0.9352	−0.0411	0.1888	0.141*	
H8B	0.8694	−0.0961	0.0761	0.141*	
H8C	0.7121	−0.0930	0.1601	0.141*	

### Atomic displacement parameters (Å^2^)

**Table:**

	*U*^11^	*U*^22^	*U*^33^	*U*^12^	*U*^13^	*U*^23^
O1	0.0377 (13)	0.0655 (17)	0.0879 (19)	0.0015 (11)	0.0087 (12)	0.0270 (14)
O2	0.0510 (15)	0.0536 (15)	0.103 (2)	−0.0012 (12)	0.0227 (14)	0.0214 (15)
C1	0.0434 (19)	0.0489 (19)	0.0519 (19)	−0.0079 (15)	0.0049 (14)	−0.0012 (15)
C2	0.0377 (16)	0.0466 (18)	0.0418 (16)	−0.0111 (13)	0.0013 (13)	−0.0047 (14)
C3	0.0415 (17)	0.053 (2)	0.0490 (18)	−0.0096 (15)	0.0093 (14)	0.0067 (15)
C4	0.0342 (16)	0.058 (2)	0.0500 (18)	−0.0045 (14)	0.0094 (13)	0.0012 (16)
O3	0.0704 (18)	0.0558 (17)	0.137 (3)	0.0022 (14)	0.0415 (19)	0.0251 (18)
N1	0.0621 (18)	0.0444 (16)	0.0636 (19)	−0.0028 (14)	0.0010 (15)	0.0095 (14)
C5	0.167 (6)	0.094 (4)	0.070 (3)	0.034 (4)	0.024 (3)	−0.002 (3)
C6	0.100 (3)	0.071 (3)	0.062 (2)	0.019 (2)	0.004 (2)	0.010 (2)
C7	0.058 (2)	0.072 (3)	0.066 (2)	−0.0090 (19)	0.0050 (18)	0.002 (2)
C8	0.100 (4)	0.073 (3)	0.111 (4)	0.015 (3)	0.020 (3)	−0.006 (3)

### Geometric parameters (Å, °)

**Table:**

O1—C1	1.245 (4)	N1—H1	0.9100
O2—C1	1.252 (4)	C5—C6	1.442 (6)
C1—C2	1.502 (5)	C5—H5A	0.9600
C2—C4	1.384 (4)	C5—H5B	0.9600
C2—C3	1.388 (4)	C5—H5C	0.9600
C3—C4^i^	1.368 (5)	C6—H6A	0.9700
C3—H3A	0.9300	C6—H6B	0.9700
C4—C3^i^	1.368 (5)	C7—C8	1.481 (6)
C4—H4	0.9300	C7—H7A	0.9700
O3—N1	1.408 (4)	C7—H7B	0.9700
O3—H3	0.8200	C8—H8A	0.9600
N1—C7	1.484 (5)	C8—H8B	0.9600
N1—C6	1.494 (5)	C8—H8C	0.9600
			
O1—C1—O2	123.8 (3)	C6—C5—H5C	109.5
O1—C1—C2	117.9 (3)	H5A—C5—H5C	109.5
O2—C1—C2	118.3 (3)	H5B—C5—H5C	109.5
C4—C2—C3	118.3 (3)	C5—C6—N1	111.9 (4)
C4—C2—C1	120.8 (3)	C5—C6—H6A	109.2
C3—C2—C1	120.9 (3)	N1—C6—H6A	109.2
C4^i^—C3—C2	121.0 (3)	C5—C6—H6B	109.2
C4^i^—C3—H3A	119.5	N1—C6—H6B	109.2
C2—C3—H3A	119.5	H6A—C6—H6B	107.9
C3^i^—C4—C2	120.7 (3)	C8—C7—N1	114.3 (4)
C3^i^—C4—H4	119.7	C8—C7—H7A	108.7
C2—C4—H4	119.7	N1—C7—H7A	108.7
N1—O3—H3	109.5	C8—C7—H7B	108.7
O3—N1—C7	108.7 (3)	N1—C7—H7B	108.7
O3—N1—C6	113.4 (3)	H7A—C7—H7B	107.6
C7—N1—C6	111.6 (3)	C7—C8—H8A	109.5
O3—N1—H1	107.7	C7—C8—H8B	109.5
C7—N1—H1	107.7	H8A—C8—H8B	109.5
C6—N1—H1	107.7	C7—C8—H8C	109.5
C6—C5—H5A	109.5	H8A—C8—H8C	109.5
C6—C5—H5B	109.5	H8B—C8—H8C	109.5
H5A—C5—H5B	109.5		
			
O1—C1—C2—C4	11.2 (5)	C3—C2—C4—C3^i^	0.6 (5)
O2—C1—C2—C4	−169.9 (3)	C1—C2—C4—C3^i^	−178.4 (3)
O1—C1—C2—C3	−167.8 (3)	O3—N1—C6—C5	−71.7 (5)
O2—C1—C2—C3	11.1 (5)	C7—N1—C6—C5	165.2 (4)
C4—C2—C3—C4^i^	−0.6 (5)	O3—N1—C7—C8	−62.6 (4)
C1—C2—C3—C4^i^	178.4 (3)	C6—N1—C7—C8	63.1 (5)

Symmetry codes: (i) −*x*, −*y*+1, −*z*.

### Hydrogen-bond geometry (Å, °)

**Table:**

*D*—H···*A*	*D*—H	H···*A*	*D*···*A*	*D*—H···*A*
O3—H3···O2	0.82	1.78	2.576 (5)	164
N1—H1···O1	0.91	1.72	2.605 (5)	164
C7—H7b···O2^ii^	0.97	2.42	3.327 (5)	156

Symmetry codes: (ii) *x*+1, *y*, *z*.
